# Variation in synonymous evolutionary rates in the SARS-CoV-2 genome

**DOI:** 10.3389/fmicb.2023.1136386

**Published:** 2023-03-09

**Authors:** Qianru Sun, Jinfeng Zeng, Kang Tang, Haoyu Long, Chi Zhang, Jie Zhang, Jing Tang, Yuting Xin, Jialu Zheng, Litao Sun, Siyang Liu, Xiangjun Du

**Affiliations:** ^1^School of Public Health (Shenzhen), Shenzhen Campus of Sun Yat-sen University, Shenzhen, China; ^2^School of Public Health (Shenzhen), Sun Yat-sen University, Guangzhou, China; ^3^Key Laboratory of Tropical Disease Control, Ministry of Education, Sun Yat-sen University, Guangzhou, China

**Keywords:** binding motif, codon usage, dominant variants, SARS-CoV-2, synonymous evolutionary rate

## Abstract

**Introduction:**

Coronavirus disease 2019 is an infectious disease caused by severe acute respiratory syndrome coronavirus 2 (SARS-CoV-2). Influential variants and mutants of this virus continue to emerge, and more effective virus-related information is urgently required for identifying and predicting new mutants. According to earlier reports, synonymous substitutions were considered phenotypically silent; thus, such mutations were frequently ignored in studies of viral mutations because they did not directly cause amino acid changes. However, recent studies have shown that synonymous substitutions are not completely silent, and their patterns and potential functional correlations should thus be delineated for better control of the pandemic.

**Methods:**

In this study, we estimated the synonymous evolutionary rate (SER) across the SARS-CoV-2 genome and used it to infer the relationship between the viral RNA and host protein. We also assessed the patterns of characteristic mutations found in different viral lineages.

**Results:**

We found that the SER varies across the genome and that the variation is primarily influenced by codon-related factors. Moreover, the conserved motifs identified based on the SER were found to be related to host RNA transport and regulation. Importantly, the majority of the existing fixed-characteristic mutations for five important virus lineages (Alpha, Beta, Gamma, Delta, and Omicron) were significantly enriched in partially constrained regions.

**Discussion:**

Taken together, our results provide unique information on the evolutionary and functional dynamics of SARS-CoV-2 based on synonymous mutations and offer potentially useful information for better control of the SARS-CoV-2 pandemic.

## Introduction

Since its first appearance 3 years ago, coronavirus disease 2019, which is caused by severe acute respiratory syndrome coronavirus 2 (SARS-CoV-2), has been declared a global pandemic, and influential variants continue to emerge and spread globally. For better monitoring and research (Tao et al., 2021; Kumar et al., 2022), the World Health Organization has listed some of the key viral variants or lineages with important mutations as variants of concern. Variants with different mutation combinations can emerge within short periods and have different effects. Therefore, it is crucial to understand this process from the evolutionary perspective for better prevention and control of the epidemic. Currently, whether a mutation is deleterious is primarily determined by comparing the relevant lineage with reference sequences through multiple sequence alignment or evidence from biological experiments (Badua et al., 2021; Lauring and Hodcroft, 2021). For example, Nextstrain uses the number of mutations at each site or the entropy of change to represent the site's degree of variability based on phylogenetic trees and some viral infection experiments assessing specific mutations (Hadfield et al., 2018; Zhang L. et al., 2020; Motozono et al., 2021; Tao et al., 2021). However, whether new mutants truly increase virus transmissibility and infectivity depends not only on the accumulation of mutations but also on the recurrence or rapid removal of mutations and their epistatic effects. Traditional analytical methods based on fixed mutations can elucidate the importance of mutations; however, owing to the time-consuming experimental verification and the rapidity of viral mutations, new methods are warranted for better and timely acquisition of updated critical information.

The *dN/dS* (K_a_/K_s_) value, where *dN* or *K*_*a*_ represents the number of non-synonymous substitutions/number of non-synonymous sites and *dS* or *K*_*s*_ represents the number of synonymous substitutions/number of synonymous sites, is always used to determine whether there is evidence for the selection of species, lineages, or proteins and gene areas (Duffy et al., 2008; Wilson et al., 2020; MacLean et al., 2021). In reality, the majority of the observed mutations are a result of natural selection and genetic drift. The aforementioned *dN/dS* indicator can also be used to determine the direction of selection. *dN* is more impacted by natural selection because amino acid alterations are always generated through selection; by contrast, *dS* is more related to the background mutation rates because such mutations do not directly cause amino acid changes. However, whether synonymous mutations represent the complete viral background remains a matter of debate in recent years. Some studies have suggested that a substantial proportion of synonymous alterations are not silent; selection, codon usage, and other factors can influence synonymous variations (de Oliveira et al., 2021; Mordstein et al., 2021; Rahman et al., 2021; Shen et al., 2022). However, it remains unclear how data on synonymous mutations in the SARS-CoV-2 genome can offer additional, in-depth knowledge on evolutionary processes and inform rules and guidelines for the precise prevention and control of the pandemic.

Furthermore, a viral infection of host cells is a complex, multistep, and often specific process. Like other RNA viruses, SARS-CoV-2 relies on regulators to effectively utilize host cellular factors at many biochemical levels, including RNA stability, processing, localization, and translation, to facilitate replication and progeny production (Flynn et al., 2021). Although existing studies have explored the proteins that can bind viral RNA and their downstream regulatory metabolic pathways from the host's perspective (Flynn et al., 2021; Khan et al., 2021; Schmidt et al., 2021), the viral genome is known to mutate faster than the host genome. This feature jeopardizes the efficacy of vaccines and drugs. Moreover, different regions of viral genomes evolve at different rates, with some regions being hypervariable and others being conserved. Until now, few studies have assessed the conservation of the virus and its relationship with the interaction patterns between viruses and hosts, especially from the perspective of synonymous mutations; more studies are needed to explore this further.

Based on the foregoing questions, it is important to explore the synonymous evolutionary rate (SER) in the open reading frames (ORFs) of the SARS-CoV-2 genome, the factors that influence the SER, and what rules can be drawn through comparison of fixed-characteristic amino acid mutations with different lineages. To answer these questions, using a mutation network approach (Zhang C. et al., 2020; Wang Y. et al., 2021), we described the distribution of the SER across the SARS-CoV-2 genome along with its influential factors and explored the conserved motifs based on the SER and the motifs' potential functional relationships with the host by performing enrichment analyses. We also assessed the potentially important and functional amino acid mutations based on the SER for identifying future dominant variants to better control the pandemic.

## Materials and methods

### Sequence data

A total of 2,537,286 original SARS-CoV-2 genomic sequences were downloaded from the Global Initiative on Sharing All Influenza Data system (Elbe and Buckland-Merrett, 2017; Shu and McCauley, 2017; Khare et al., 2021) as of 15 September 2021. Sequences were excluded if they met any of the following criteria: (1) genome size of <29,000 nucleotides; (2) >5% of undetermined nucleotides; (3) non-human host. To further ensure sequence quality, sequences with complete collection date, region details (specific to the country), and a gap length of <400 bp were included. The sequences were first aligned using MAFFT v7.310 (Katoh and Standley, 2013), with Wuhan-Hu-1 (MN908947.3) as the reference. The alignment command was as follows: *mafft-*−*6merpair—thread-12—keeplength—addfragments othersequences referencesequence* > *output*. Moreover, the redundant sequences, which are sequences with identical nucleotide compositions, were filtered out; however, the redundant sequence with the earliest collection time was included because the connected edges of the mutation network are based on the mutation probability. If two sequences were the same (without any mutation), the probability between them was 1. Therefore, we believed that only transmission and no evolution occurred between the two sequences and that they could not provide more evolutionary information. Next, we conducted stratified sampling per country (region) per day. Finally, a total of 10,089 sequences were included in this study (accessible at 10.55876/gis8.230130ru; also, in Supplementary Table 7). We also masked the problematic sites to avoid artificial errors using the methods outlined at https://virological.org/t/masking-strategies-for-sars-cov-2-alignments/480 (Oliver et al., 2021). Finally, we filled the undetermined nucleotides or gaps with the element with the highest frequency at the corresponding position based on the top 10 closest sequences measured by the Hamming distance (Wang Y. et al., 2021). Subsequently, except for stop codons and non-coding sites, sites corresponding to protein-coding ORFs were mapped to the reference sequence alignment and eventually used to construct the mutation network.

### SER estimation

Following the methods outlined by Zhang C. et al. (2020), a directed and weighted mutation network was constructed with the nodes representing strains and links which represent pairs of strains, and a mutation probability of no more than the predetermined threshold (>10th percentile). The baseline mutation probabilities among A, T, G, and C were extracted from pairs of sequences with single-nucleotide differences in the corresponding data. The mutation probabilities between pairs of strains with different numbers of mutations were calculated as the product of probabilities of the single mutations (Zhang C. et al., 2020; Wang Y. et al., 2021) (Supplementary Figure 1). Paths on the network were extracted using the random walk method. First, 20,000 start nodes were randomly chosen that have descendants (out-degree ≠ 0), and second, a random walk was executed from selected start nodes. The paths yielded by the repeated random walk were considered evolutionary paths in the real world. Moreover, the nodes on the path had an evolutionary ancestor–descendant relationship. To ensure sufficient divergence, only paths with more than 1 month were included. Python package NetworkX v2.8.4 was used for the analysis (Hagberg et al., 2008). In summary, for the final mutation network, the input was 10,089 sequences and the output was the evolutionary paths got from random walks.

Different ORFs in the SARS-CoV-2 genome have different lengths (Supplementary Table S1). To avoid biases caused by the ORF length, we used a codon-based sliding window approach; a 600-bp window and 3-bp step were maintained. The 600-bp window was set after considering the upper limit of the substitution rate of the virus to ensure sufficient observation of substitutions along any chosen path. The KaKs_Calculator v2.0 software MLWL model was used to calculate the *dS* value (Tzeng et al., 2004; Wang et al., 2010). The following command was used: *KaKs_Calculator -i input -o out -m MLWL*. Next, a linear regression analysis of *dS* on the collection time interval was performed, and the regression line slope was represented as the SER for the start position of the window (Ho and Duchene, 2014; Kim et al., 2022). The Kruskal–Wallis test and Mann–Whitney U-test were used to compare the statistical differences between the ORFs. Based on the SER (10th percentile, 50th percentile, and 90th percentile), the genome was divided into four regions: (1) the free region (the region with an upper 90th percentile SER); (2) the slightly free region (the region with an SER between the 50th percentile and 90th percentile); (3) the partially constrained region (the region with an SER between the 10th percentile and 50th percentile); and (4) the constrained region (the region with an SER lower than the 10th percentile SER).

### Motif identification and function association analysis

With the constrained regions set as the target and three other groups set as the background, we used STREME v5.5.0 and a zero-order Markov model for background model creation in the MEME suite server to find conserved sequence patterns (motifs) with a sequence length of 3–30 bp (Bailey et al., 2015; Bailey, 2021), a *P*-value of <0.001, and coverage of >70%. Next, we used the find individual motif occurrence (FIMO v5.5.0) program to locate the motif position with a *P*-value of <1e−4 for double chains in the sequence (Grant et al., 2011).

The RNA motif data recognized by RNA-binding proteins (RBPs) were obtained from a previous study (Ray et al., 2013); only records from *Homo sapiens* were included. The Tomtom motif comparison tool v5.5.0 in the MEME suite server is used to compare motifs against a database of known motifs. In this study, we used this tool to compare motif similarity and identify host-associated proteins with default settings (Gupta et al., 2007). Cytoscape v3.8.0 was used to visualize the protein–motif relationships (Shannon et al., 2003). We also conducted Gene Ontology (GO) enrichment analysis based on the hypergeometric distribution using clusterProfiler v4.6.0 package in R with default parameters (Yu et al., 2012).

### Feature collection and model construction

To determine the dinucleotide composition (CpG and UpA), we divided the dinucleotide frequency within the sequence by the product of the frequency of each nucleotide (Mordstein et al., 2021). All codon usage index types, including the codon bias index, the effective number of codons, GC content and GC content in the third codon (GC, GC3), and silent base composition (A3, T3, G3, and C3), were calculated using CodonW v1.4.4 with default parameters (Peden, 2000); the protein hydrophobicity was also calculated using CodonW. The ω (*dN/dS*) value, which represents the selection of entire ORFs, was estimated using the BUSTED method in HyPhy v2.5.2 with default parameters (Murrell et al., 2015). By contrast, the non-synonymous evolutionary rate (NER), which represents the selection in codon sites, was calculated similarly to SER by fitting the regression line of *dN* and the collection time interval. The normalized van der Waals volume and relative mutability for each window were extracted and calculated using the AAindex2 database (Kawashima et al., 2008). The minimum free energy of the RNA secondary structure in the windows was determined using RNAstructure Fold server v6.4 with the default parameters (Reuter and Mathews, 2010). Based on the absolute difference between the two sequences, the aforementioned features were used for the following analysis: for motif information, “0” was assigned if the motif did not exist; “1” was assigned if the motif existed in one sequence; and “2” was assigned if the motif existed in both sequences.

Features were filtered based on the results of Spearman's correlation analysis. Based on the aforementioned features, a light gradient-boosting machine (LightGBM) regression model was constructed for determining the SER, and R-squared values were used to measure any explicable variations (Meng and Liu, 2017). Next, 80% of the randomly selected data were used as the training set, and the remaining 20% were set as the test set. The GridSearchCV technique and 10-fold cross-validation were employed to determine the best hyperparameters for model construction (Pedregosa et al., 2011). Subsequently, the SHapley Additive exPlanations (SHAP) value was used to explain the output of the constructed machine learning model to evaluate feature importance (Lundberg et al., 2020). The feature value represents the value of each feature in the model, ranging from small to large and from blue color to red. The SHAP value represents the direction and size of the SER affected by each sample; a value >0 indicates a positive impact, and any other value indicates a negative impact. LightGBM v3.3.3, scikit-learn v1.0.2, and shap v0.41.0 packages were used for these analyses.

### Comparison of fixed-characteristic mutations in different lineages

Fixed-characteristic amino acid mutations, including deletions accumulated in different lineages, were downloaded from the Cov-Lineages repository (https://cov-lineages.org/lineage_list.html). Characteristic mutations in the lineages Alpha, Beta, Gamma, Delta, and Omicron (sub-lineages: BA.1, BA.2, BA.2.12.1, BA.2.75, BA.4, and BA.5) were used in our analysis (Supplementary Table 6; **Figure 4C**).

### Statistical analysis

The Kruskal–Wallis, Mann–Whitney U, and chi-square tests (α = 0.05) were used with the stats.kruskal function, stats.mannwhitneyu function, and stats.chi2_contigency function, respectively, in SciPy 1.5.2 package in Python 3.8.5. Furthermore, the ggplot2 3.3.5 package in R 4.1.1 and matplotlib 3.3.2 in Python 3.8.5 were used to generate most figures.

## Results

### SER landscape for the SARS-CoV-2 genome

We constructed the mutation network such that it was scale-free (Supplementary Figure 2). Based on the created mutation network, random walks were executed 20,000 times, and the potential paths between sequence pairs were extracted. Because of the strong similarities among SARS-CoV-2 viruses, only paths between paired nodes with a time interval of >1 month were included in the following analysis.

**Figure 1:**
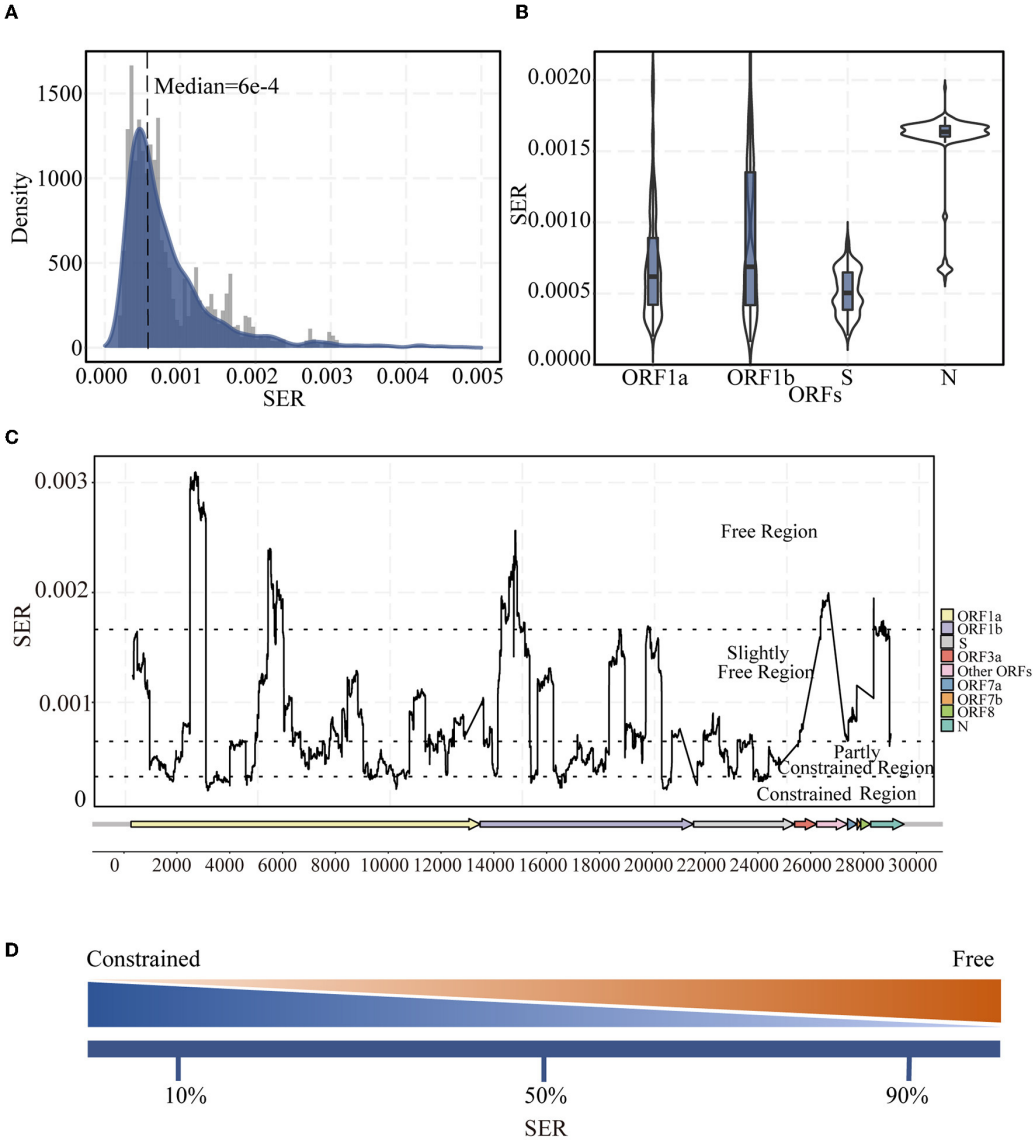
Landscape of synonymous evolutionary rate (SER) of the SARS-CoV-2 genome. **(A)** SER density distribution in all ORFs of SARS-CoV-2. **(B)** Violin plot of SER distribution for representative ORF1a, ORF1b, S, and N regions. **(C)** SER across the whole genomes based on sliding windows. Black dotted lines were 90th, 50th, and 10th percentile levels of SER. **(D)** Percentiles are used to divide regions. The greater the SER, the more freedom; the smaller the SER, the greater the constraint.

In general, the SER distribution across the whole genome was extremely skewed and lopsided, displaying the characteristics of Gamma distribution, with a median (Q1, Q3) of 6 × 10^−4^ (4 × 10^−4^, 1.1 × 10^−3^) per site per year across all regions (Figure 1A). The SER was highly variable, with averages ranging from 5 × 10^−4^ to 2 × 10^−3^ per site per year (Figure 1B). Moreover, the SERs of different ORFs (*H* = 982.1478, *P* < 0.001) and between any of the ORFs (adjusted *P* < 0.05) were significantly different. The SERs within the SARS-CoV-2 genome were also substantially different (Figure 1C). The fluctuations were obvious, as indicated through traditional diversity cues, implying that the SERs varied widely and the synonymous substitutions tended to be enriched or reduced in specific genomic regions. Based on the SERs (10th percentile, 50th percentile, and 90th percentile), the genome was divided into four regions, as explained in the Methods section (Figures 1A, D). The overall SER for the S gene was low and mostly located within the partly constrained region (Figure 1C), which was different from that identified in the traditional diversity analysis (Supplementary Figure 3). This difference was not caused by the increased NER (Supplementary Figure 4). Moreover, the SER in the ORF1ab region tended to have more freedom toward a greater variation.

### Characteristics of the conserved motifs in the constrained region

**Figure 2:**
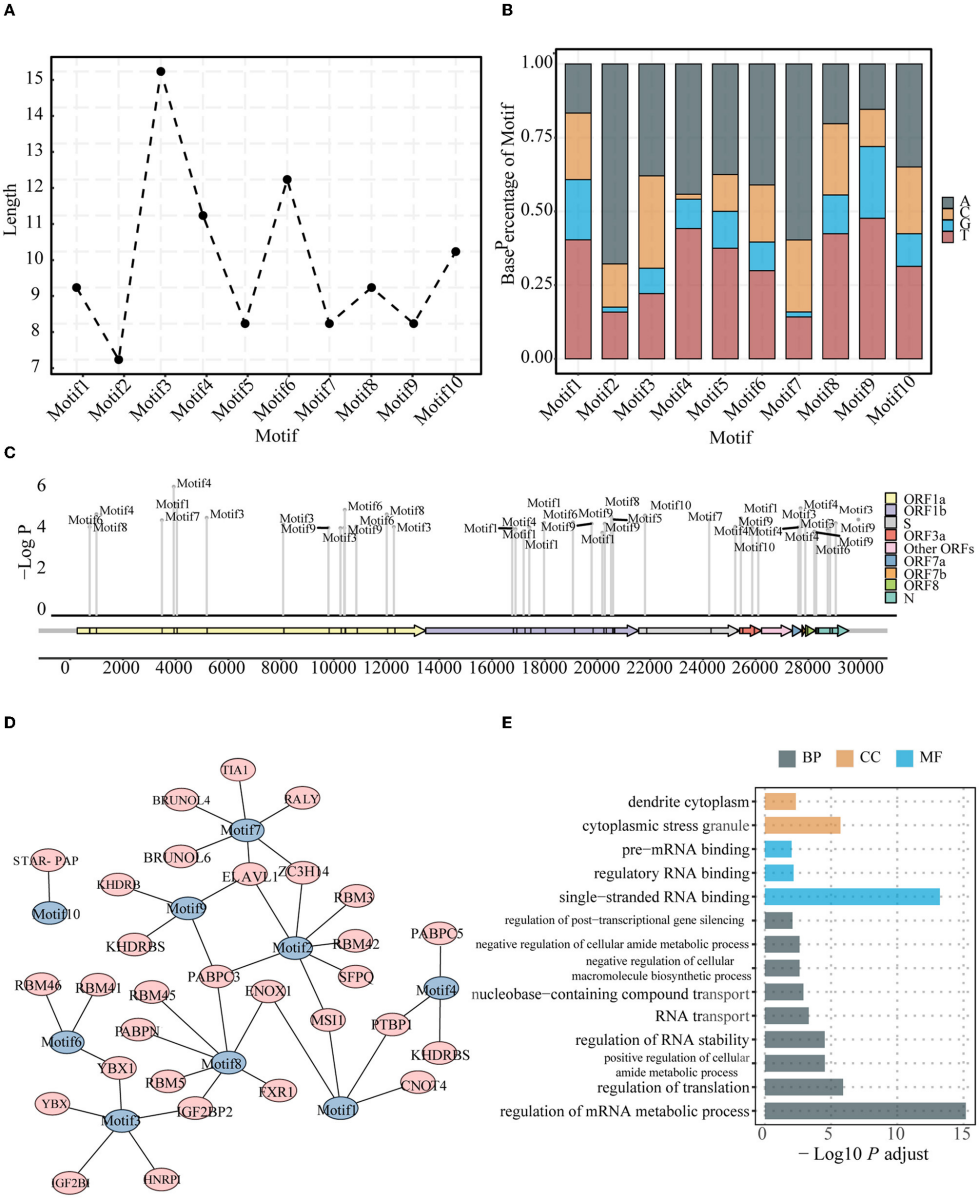
Characteristics for the conserved motifs. Length **(A)** and Base composition **(B)** of identified motifs enriched in the constrained regions. The identification indexes are defined by sorting by *P*-value from the smallest to the largest. **(C)** Positions of identified motifs on the genome. The location of identified motifs was indicated by short black blocks in the ORFs. The vertical axis represents the credibility of the motif. **(D)** Motifs and related human RBPs. Motifs are colored in blue, and RBPs are colored in red. **(E)** GO terms enriched for motif-related human RBPs, including biological process, molecular function, and cellular component.

To check whether conserved sequences (motifs) existed in the constrained region (Supplementary Table 2), we performed an enrichment analysis for comparing sequences in the constrained region using other regions as the background. After strict filtering, we obtained 10 motifs with a length of ~9–15 bp (Figure 2A; Supplementary Table 8). The Kruskal–Wallis test results indicated that the base composition was statistically significant and that the A + T content in the motifs was higher than the G + C content (*P* = 6e−4) (Figure 2B). Furthermore, these motifs were found in various ORFs throughout the genome (Figure 2C).

Previous studies have revealed that some regions of the viral genome are preferred by host proteins (Flynn et al., 2021; Khan et al., 2021; Lu et al., 2021; Schmidt et al., 2021). In other words, the host RBPs could specifically bind certain sequences such as motifs on the viral genome. The identified motifs from the viral genome were thus compared with some known binding motifs of the host RBPs. A total of 30 host protein genes were found to be associated with the 10 identified motifs (Figure 2D). Of note, some motifs may be targeted by more than one host protein, and the same host protein may bind different motifs in the viral genome. Remarkably, *YBX1*, which was identified to bind Motifs3 and Motif6, was found to be associated with viral infections, including SARS-CoV-2 and Zika, and previous experiments have shown that knockout of this gene can reduce the infection intensity (Zhang et al., 2022). Some other associated host proteins were also found in some experimental studies assessing viral infection; for example, *SFPQ* was found to interact with the SARS-CoV-2 genome and promote viral RNA amplification (Labeau et al., 2022). Functional GO annotation revealed that these genes are involved in metabolic RNA regulation (Figure 2E).

### Factors contributing to the SER variations

To further investigate the factors that may contribute to the variations in the SERs in the SARS-CoV-2 genome, the codon usage index, the dinucleotide composition, the selection index, the structure index, and the motif information were included and fed into the model. The features were classified into five groups: the codon usage index, selection index, dinucleotide composition, structure index (Resch et al., 2007; Callens et al., 2021; McGrath, 2021; Mordstein et al., 2021; Pintó and Bosch, 2021), and conserved motifs were identified in this study (Supplementary Table 3). G3, gravy, *van der Waals* volume, and aa mutations were excluded owing to high collinearity based on the correlation coefficients (*R*^2^ > 0.9, Supplementary Figure 5), whereas the other features were included and used in the LightGBM model.

**Figure 3:**
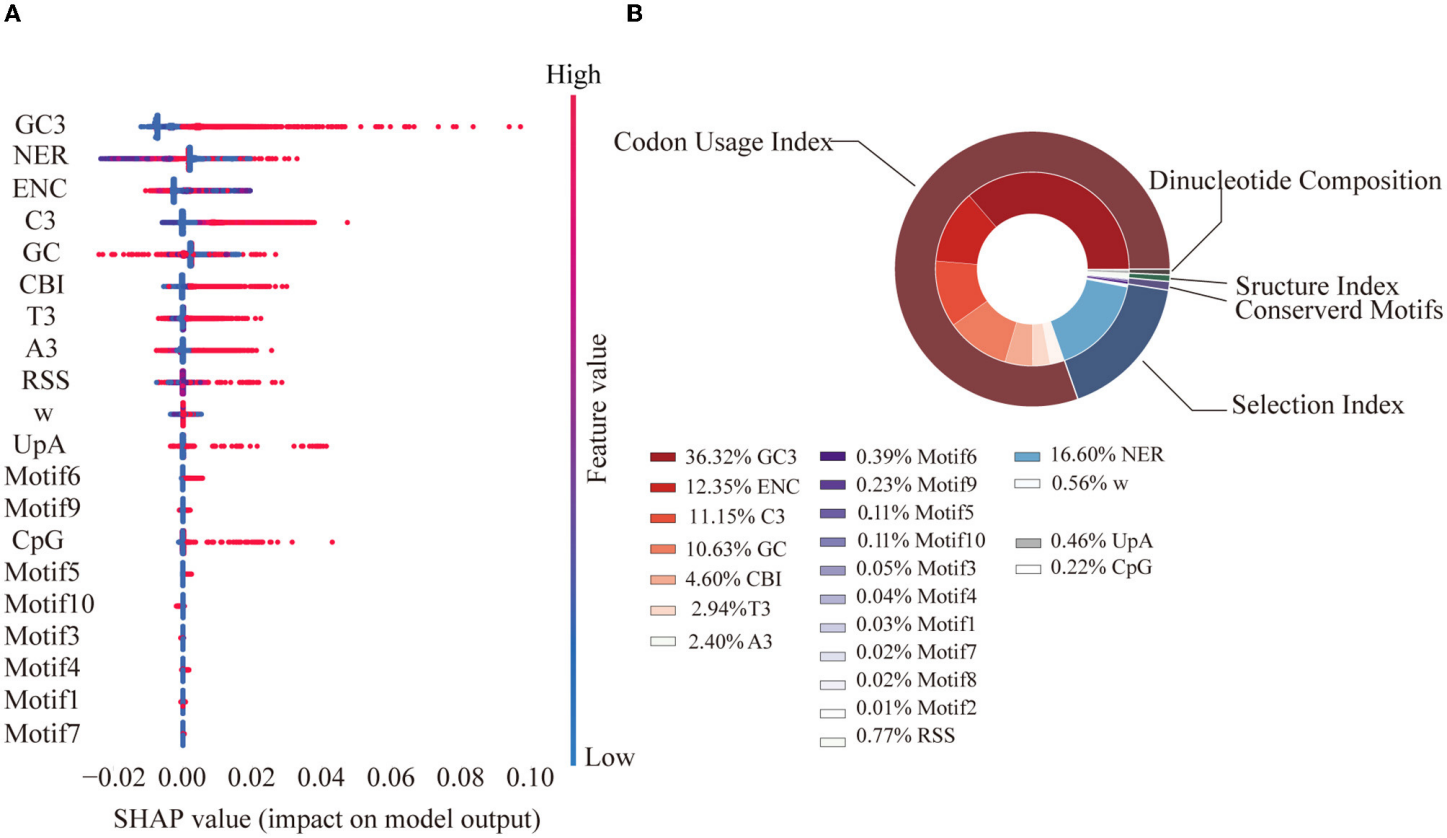
Feature SHAP value and contribution. **(A)** SHAP value for the top 20 features. Each point represents a sample. A SHAP value greater than 0 contributes to a higher SER, while a value less than 0 contributes to a lower SER. Feature value represents the value of each sample. **(B)** Feature importance pie chart. The outer ring represents the grouping, while the inner ring represents each specific feature. Percentage represents the proportion of the total interpretability.

Based on cross-validation, the best model after grid search (Supplementary Table 4) had an adjusted R^2^ of 0.72 on the training dataset and 0.69 on the test dataset, indicating good performance (Supplementary Table 5). According to the final model, factors from the codon usage index group contributed the most to the variations in the SERs (80.37%). GC3 (36.32%) was the most important single feature, followed by the non-SER (16.60%) from the group of selection (Figure 3).

### Association between the accumulated characteristic mutations and SERs

**Figure 4:**
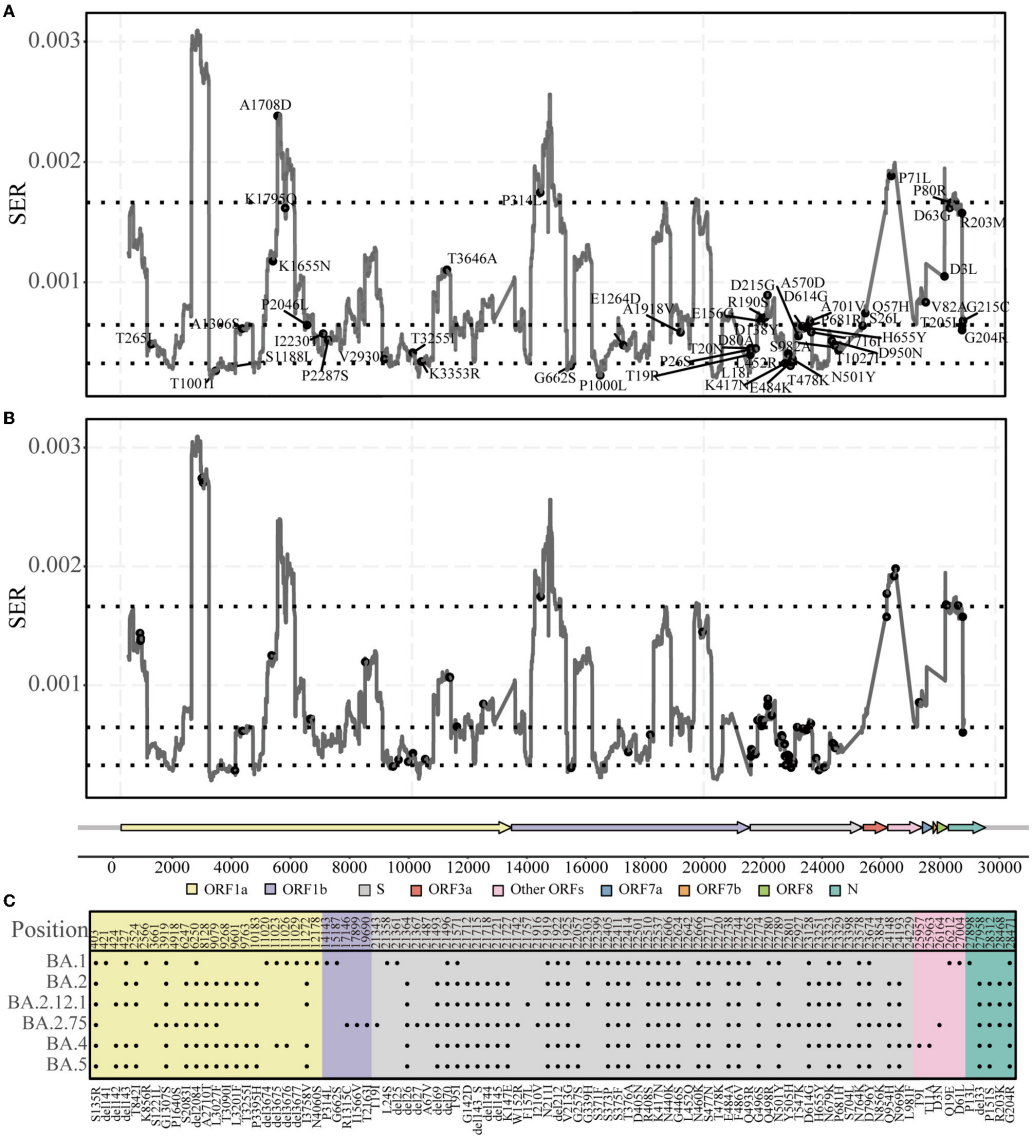
Characteristic mutations in five lineages. **(A)** Accumulated characteristic mutations in Alpha, Beta, Gamma, and Delta lineages. **(B, C)** Accumulated characteristic mutations in the Omicron lineage and their specific positions.

The characteristic mutations accumulated in the five main lineages (Alpha, Beta, Gamma, Delta, and Omicron) were mapped onto the SER landscape of the SARS-CoV-2 genome to investigate their associations (Figure 4). Based on the classification of the four regions across the genome based on the SER landscape, because most mutations exist in the middle region, the chi-square test was used to compare the number of characteristic mutations between the middle two groups, and the total number of positions in the two groups was found to be consistent and comparable. From a statistical viewpoint, the results of the four lineages that appeared first (Alpha, Beta, Gamma, and Delta) and were used to estimate the SER herein revealed that the characteristic mutations were significantly preferred in the partially constrained region than in the slightly free region (adjusted *chi-square, P* = 0.036) (Table 1). For the Omicron lineages, the sequences of which were not included in the SER estimation, characteristic mutations from the BA.2, BA.2.12.1, BA.4, and BA.5 sub-lineages showed a significant preference in the partially constrained regions, whereas the trend was not significant for BA.1 (adjusted *chi-square, P* = 0.449). For BA.2.75, a marginal *P*-value of 0.054 was obtained, indicating insufficient significance.

**Table 1:** Statistical test for positions of characteristic mutation accumulated in lineages.

**Region**	**Number of characteristic mutations**	**Number of not characteristic mutations**	*P* value^*^
**Alpha, Beta, Gamma and Delta**			
Slightly free region	15	3,307	0.036
Partly constrained regions	30	3,292	
**Omicron-BA.1**			
Slightly free region	19	3,303	0.449
Partly constrained regions	25	3,297	
**Omicron-BA.2**			
Slightly free region	13	3,309	0.005
Partly constrained regions	33	3,289	
**Omicron-BA.2.12.1**			
Slightly free region	15	3,307	0.010
Partly constrained regions	34	3,288	
**Omicron-BA.2.75**			
Slightly free region	16	3,306	0.054
Partly constrained regions	30	3,292	
**Omicron-BA.4**			
Slightly free region	17	3,305	0.018
Partly constrained regions	35	3,287	
**Omicron-BA.5**			
Slightly free region	13	3,309	0.003
Partly constrained regions	34	3,288	

* Adjusted chi-square test.

## Discussion

Viral synonymous changes are considered phenotypically silent, not functionally important, and frequently ignored; however, considering the continuing emergence of variants, it is necessary to speculate the significance of each type of mutation and its functional associations from the standpoint of synonymous substitutions, which are generally less studied. In this study, we found variations in the SERs across the SARS-CoV-2 genome. These variations can be partly explained by some factors, including the codon usage index, selection index, dinucleotide composition, structure index, and conserved motifs. Relevant motifs with extremely low SERs and potential functional constraints were identified in the constrained regions. Possible RBPs and their functions were also explored. The most important factor influencing the SER is the codon usage index. Fixed amino acid mutations are more likely to occur in partially constrained regions with potentially important functions and better adaptability. Our results indicated that the synonymous changes in the SARS-CoV-2 genome are not completely random and may be impacted by some fundamental functions and linked to the adaptation of future dominant variants.

Overall, the SERs in the SARS-CoV-2 genome vary across different regions. Their substitution rates (0.4–1.0 × 10^−3^ per site per year) (Figure 1A) are slightly lower than the traditionally observed substitution rates (approximately 10^−4^-10^−3^ per site per year) based on the observed diversity (Boni et al., 2020; Chaw et al., 2020; Sharun et al., 2021; Singh and Yi, 2021), and the SER still follow the gamma distribution pattern (Kelly and Rice, 1996). The SERs were estimated using data from the first 2 years after SARS-CoV-2 infected the population. To achieve a certain level of adaptability after the virus has just infected the population, the virus will ensure a higher substitution rate than that in the equilibrium state, and this equilibrium state level may be closer to the estimated rate from synonymous sites. Statistically different SER distributions were also observed in several ORFs (Figure 1B) and different SER levels (Figure 1C) for positions. Discrepancies in the SERs between ORFs were also consistent with previous findings on *dS* estimation for other coronaviruses and SARS-CoV-2 (Singh and Yi, 2021; Wang H. et al., 2021).

In addition to the very high and very low SER values owing to the strong selection, we divided the middle 80% of the SERs into two groups. In contrast to the results obtained using traditional methods, where mutation events and entropy are considered, SER was found to be low in the S region in which diversity was previously thought to be high (compare Figure 1C and Supplementary Figure 3). The S protein is the most important surface protein in coronaviruses and is closely related to the virus infectivity and pathogenesis (Andersen et al., 2020; Li Y. et al., 2021). The S protein has important evolutionary functions and functional constraints. However, owing to host switching and the long-term arms race with the host, this region experiences a certain degree of freedom, with a lot of changes occurring when it retains its original functions. Moreover, the higher diversity in the S region when counting mutation events or entropy may also be linked to the slightly deleterious mutations, which can later be removed by purifying selection. Furthermore, these measurements of diversity do not consider the rate of changes over time. However, from the SER viewpoint, the S protein region has important functions and certain adaptabilities, mostly in the partially constrained regions. All of these observations indicate that S protein changes impact the virus and could be related to adaptation.

Viruses have a simple structure, and they interact with appropriate hosts to cause infections. The viral genome plays a significant role when infecting a host (Ma-Lauer et al., 2012; Getts et al., 2013). The characteristics of conserved motifs from the constrained regions may indicate their functional importance during their interaction with a host. When matching the binding motif sites of human RBPs (Figure 2D), the identified motifs become associated with human RBPs, and some of the associated host RBPs have been identified and studied in previous coronavirus disease 2019-related studies. The knockdown of *YBX1*, which is associated with Motif3 and Motif6, reduces the viral RNA levels in both SARS-CoV-2 and Zika virus (Zhang et al., 2022). Together with *YBX1, ELAVL1*, which is found in viral RBP interactomes of SARS-CoV-2, is an IGF2BP1-related protein and a known mRNA stabilizer in humans, contributing to the stable translation of its target genes (Zhou and Pan, 2018). *SFPQ*, which interacts with the SARS-CoV-2 genome and promotes viral RNA amplification (Labeau et al., 2022), has been experimentally proven as a host factor required for the transcription of influenza virus; this can improve the transcription efficiency of viral mRNA polyadenylation (Landeras-Bueno et al., 2011). Furthermore, several *RBM* family proteins were involved in various steps of host RNA metabolism, including splicing, transportation, translation, and stability (Li Z. et al., 2021); moreover, the RBM family proteins were associated with the motifs identified in this study (Figure 2D). Functional annotation of these genes demonstrated their roles in RNA stabilization, binding single-stranded RNA, and translation regulation (Figure 2E). Our findings related to the conserved motifs from the constrained region and their potential functional importance provide a better understanding of the complete interaction landscape between the pathogen and host and may provide useful information for identifying novel drug or vaccine targets.

The features included in our model explained 72% of the SER variation. Among all the identified factors, sequence nucleotide and codon usage preferences were found to play a significant role (Figure 3). Previous experiments in eukaryotes and prokaryotes have shown that codon usage bias is associated with gene expression and translation efficiency (Frumkin et al., 2018; Yang et al., 2019). The SARS-CoV-2 genome is AU-rich and has a clear preference for AU-rich codons over GC-rich codons; a similar trend has been observed in other coronavirus genomes, where UpA and CpG dinucleotides were strictly avoided. This may be attributable to the fact that viruses need to use host tRNA for translation and that the relative abundance of tRNAs in humans is inconsistent. Preference toward a certain nucleotide composition could improve viral translation efficiency in the host (Dilucca et al., 2020). Another explanation is that this bias may help viruses evade the innate immune response in humans (Roy et al., 2021). The significant number of synonymous transitions from C to U, which were reported in previous studies of the SARS-CoV-2 genome (De Maio et al., 2021; Morales et al., 2021) as well as observed in our study, was consistent with this phenomenon. The selection index substantially contributes to the variations in SERs (17.16%), with the single feature of the non-SER contributing the highest, indicating the importance of the contribution of selection pressure from the function requirement.

As new variants continue to emerge, previous studies have identified some characteristic mutations (including deletions) that are associated with viral transmissibility or infectivity (Bhattacharya et al., 2021; Kannan et al., 2021; Kumar et al., 2022; Papanikolaou et al., 2022). We found that the accumulated characteristic mutations mostly occurred in the partly constrained regions (Figure 4; Table 1); for example, the well-known P681H, Y505H, and E484K mutations occurred in the S region of many lineages. The location of the mutations in the partly constrained regions may play important roles; for example, they may alter the transmission rates and pathogenicity but simultaneously have the flexibility for tolerating mutations. Given that the Omicron genomes form a new monophyletic group (Kandeel et al., 2021), Omicron-related comparisons are more meaningful only when their sub-lineages are compared. For example, mutations are not significantly present in the partly constrained regions of Omicron BA.1; however, the opposite is observed for BA.2. Relevant studies have shown that BA.2 is more infectious than BA.1 (Elliott et al., 2022; Lyngse et al., 2022) and that the strains BA.2.75 and BA.2.12.1 exhibit the same phenomena as BA.2 (Table 1). These observations indicate that BA.1 may not be fully adapted as compared with the other lineages, owing to its sudden emergence. Mutations were indeed enriched in the partly constrained regions of BA.4 and BA.5. These strains are expected to become popular dominant strains and subsequently evolve into some new sub-lineages. One should pay careful attention to these sub-lineages, especially to BA.5, because the majority of their accumulated mutations have important functions. Thus, an estimate of the genomic SER can help quickly determine whether a mutation has significant impacts on circulation and could uniquely contribute toward rapid decision-making for preventing epidemics by compensating for the limitations of time-consuming laboratory tests.

Our study also has some limitations. (1) Our results are only based on the SARS-CoV-2 genome, and similar investigations in other viruses are warranted in the future. (2) The conserved motifs and their potential binding relationships with the host RBPs were mainly inferred through computational analyses, which require further experimental validation. (3) Some factors may not have been included in the SER variation analysis, which may have biased the understanding presented herein, and therefore, further investigation is warranted. (4) To identify important variants, other clues still need to be found and explored. Taken together, rather than ignoring synonymous mutations, one must pay further attention to them and explore the relationship between the synonymous mutations and other factors and the underlying mechanisms. All the relevant evidence gathered over time will ultimately help us to better prevent and control existing and future infectious diseases.

## Data availability statement

The datasets presented in this study can be found in online repositories. The names of the repository/repositories and accession number(s) can be found in the article/Supplementary material.

## Author contributions

XD conceived and designed the study and supervised the study. QS analyzed the data and drafted the manuscript. QS, JZen, and JZha collected the data. QS and JZha cleaned the data. QS, JZen, KT, HL, CZ, JZha, YX, JT, JZhe, SL, and LS commented on and revised the manuscript drafts. All authors read and approved the final report.
