# Supplementary material for: Variation in synonymous evolutionary rates in the SARS-CoV-2 genome
Source: Front Microbiol. 2023 Mar 9;14:1136386. doi: 10.3389/fmicb.2023.1136386 (PMC10034387; doi:10.3389/fmicb.2023.1136386)
Supplement: Supplementary file 1 [file Data_Sheet_1.docx]

**Supplementary Information for:**

**Variation in Synonymous Evolutionary Rates in the SARS-CoV-2 Genome**

Qianru Sun,^1,2^ Jinfeng Zeng,^1,2^ Kang Tang,^1,2^ Haoyu Long,^1,2^ Chi Zhang,^1,2^ Jie Zhang,^1,2^ Jing Tang,^1,2^ Yuting Xin,^1,2^ Jialu Zheng,^1,2^ Litao Sun,^1, 2^ Siyang Liu,^1,2^ and Xiangjun Du^*,1,2,3^

1 School of Public Health (Shenzhen), Sun Yat-sen University, Guangzhou 510275, P.R. China

2 School of Public Health (Shenzhen), Shenzhen Campus of Sun Yat-sen University, Shenzhen 518107, P.R. China

3 Key Laboratory of Tropical Disease Control, Ministry of Education, Sun Yat-sen University, Guangzhou 510030, P.R. China

***Corresponding authors:** E-mail: Xiangjun Du: duxj9@mail.sysu.edu.cn

**This file including:**

**Figure S1:** **Schematic flow chart of SER estimation method.**

**Figure S2:** **Mutation network attributes of SARS-CoV-2.**

**Figure S3: Amino acid and nucleotide diversity of SARS-CoV-2 genome.**

**Figure S4: Effect of NER on SER.**

**Figure S5:** **Correlation heatmap between continuous features and SER.**

**Table S1:** **ORF lengths for SARS-CoV-2 genome.**

**Table S2: Specific position and its ORF in SARS-CoV-2 constrained region.**

**Table S3: Contributing features used in this study.**

**Table S4:** **Model hyperparameters for LightGBM model.**

**Table S5: LightGBM model performance.**

**Table S6: Amino acid mutations from Alpha, Beta, Gamma, and Delta lineages in Table 1.xlsx.**

**Table S7: Sequence information from GISAID in Table 2.pdf.**

**Table S8: PSSM for motifs found in constrained region in Table 3xlsx.**

**Supplementary Figures**

**
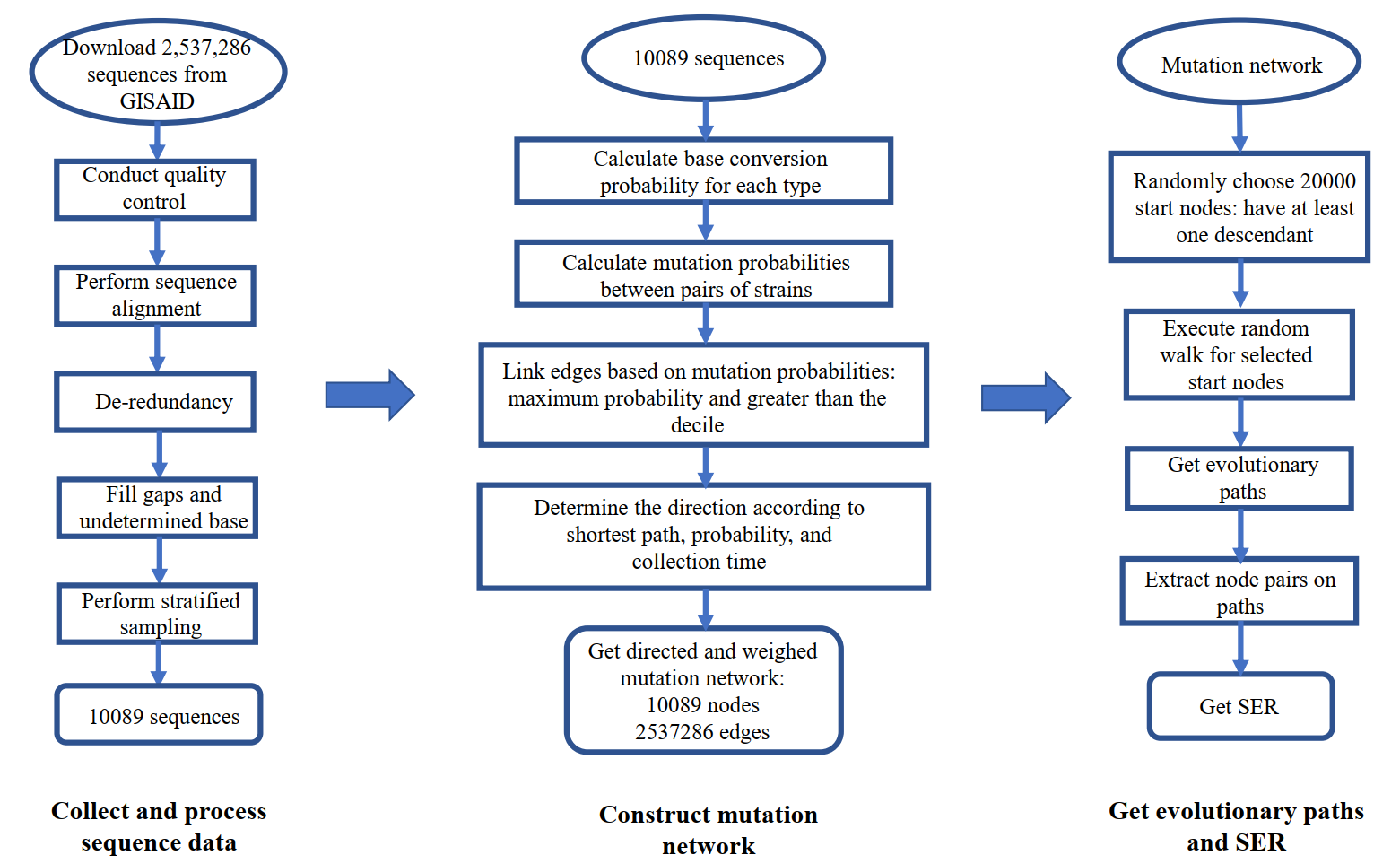
**

**Figure S1.** **Schematic flow chart of SER estimation method.**


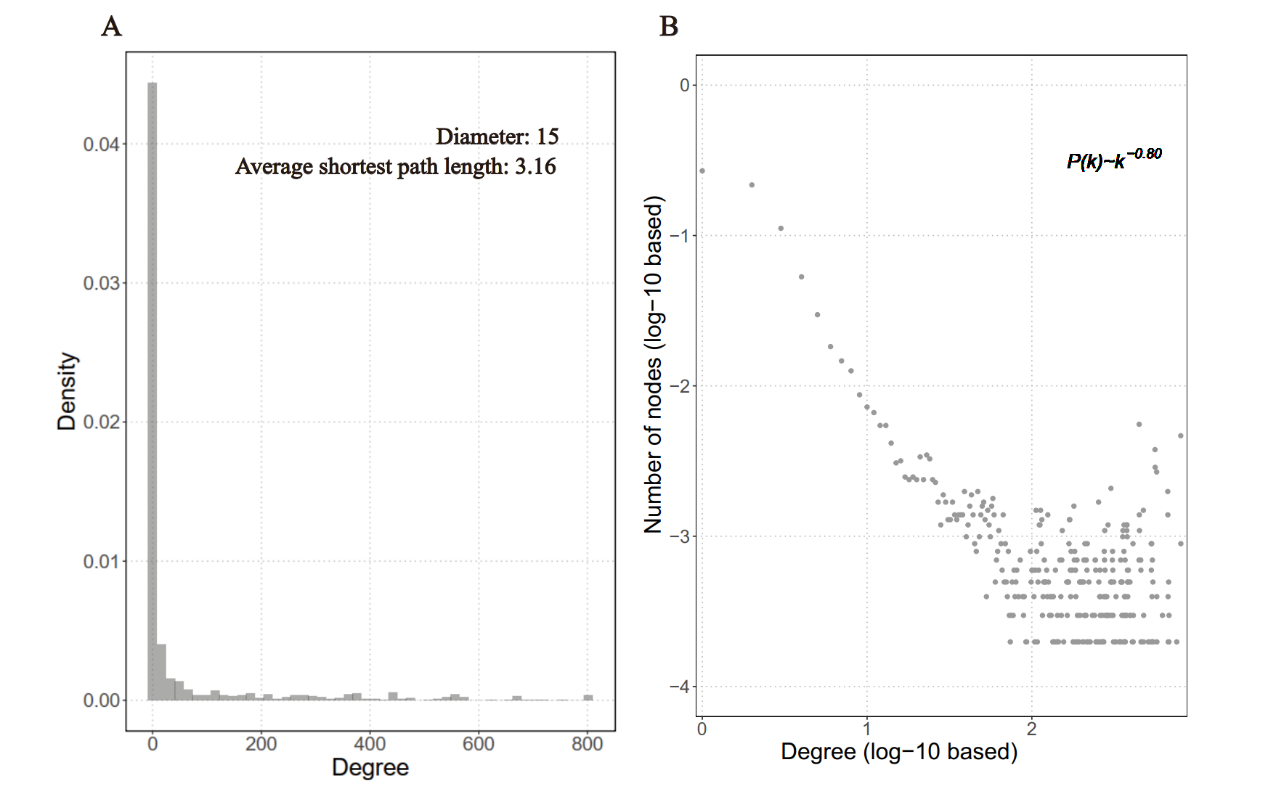


**Figure S2.** **Mutation network attributes of SARS-CoV-2.**

**(A).** Density distribution of nodes degree.

**(B).** The degree distribution of the mutation network. The power exponent in the figure reflects the slope of the linear equation, the horizontal axis is the degree under the log-10 scale, and the vertical axis is the nodes number of each degree under the log-10 scale.


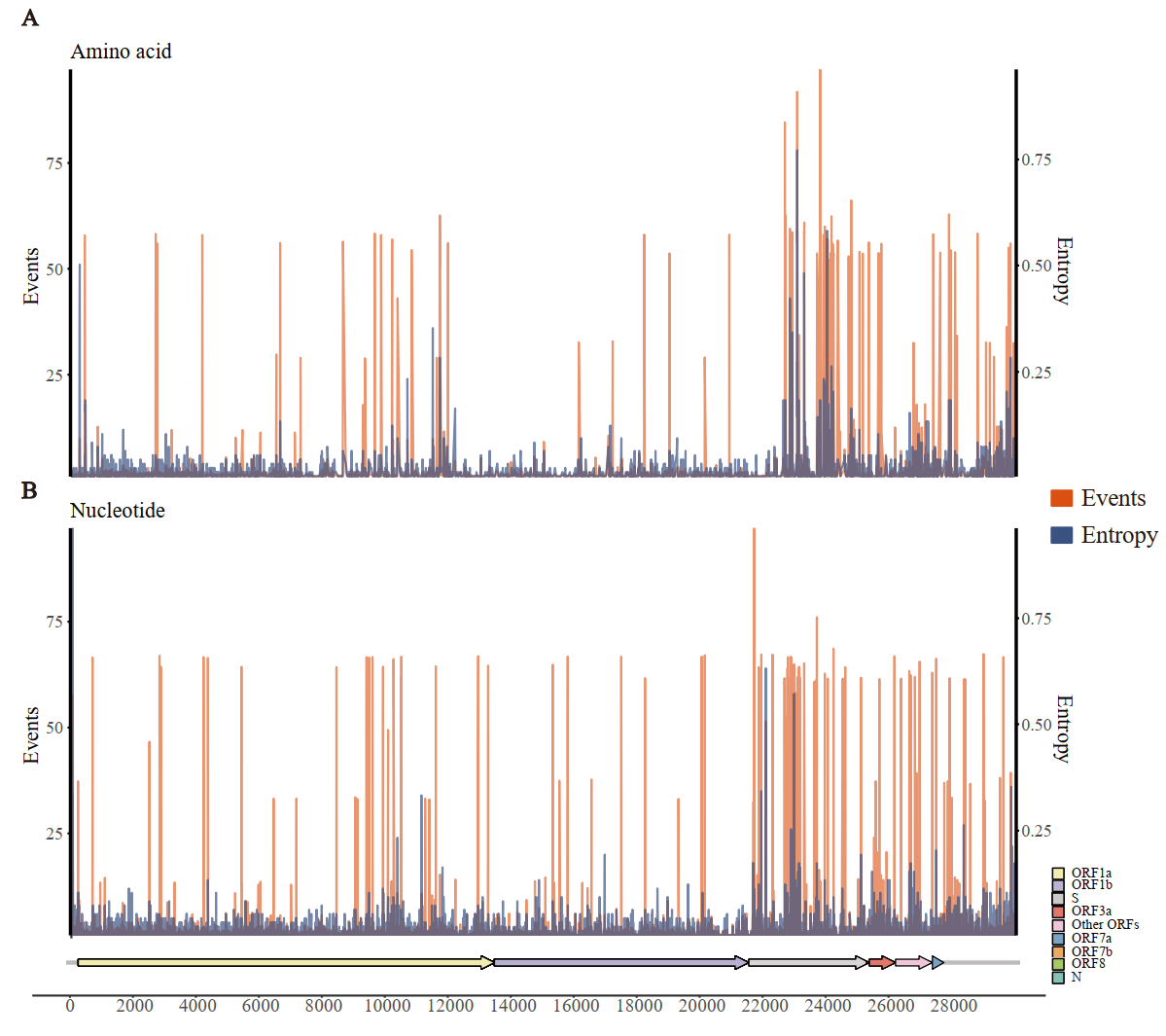


**Figure S3.** Amino acid **(A)** and nucleotide **(B)** diversity of SARS-CoV-2 genome from the Nextstrain based on both mutation events (red) and entropy (blue).


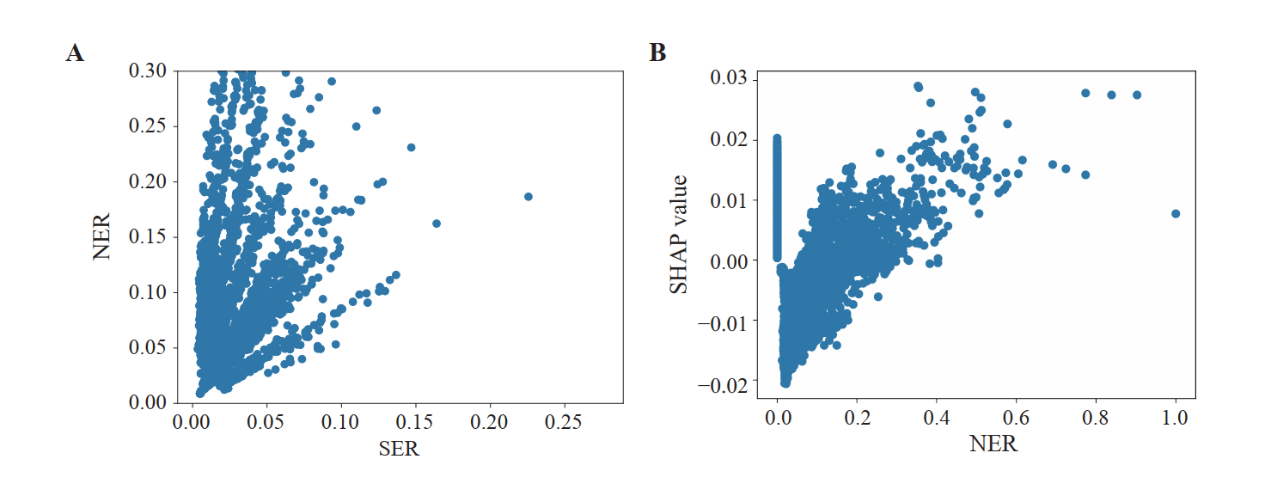
**Figure S4. Effect of NER on SER.**

**(A).** Scatter plot of NER with SER. As SER increases, so does NER, and the magnitude is greater than SER.

**(B).** Scatter plot of NER and its SHAP value. A SHAP value greater than 0 indicates a positive effect on SER. It can be shown that when NER increases, so does its positive effect on SER.


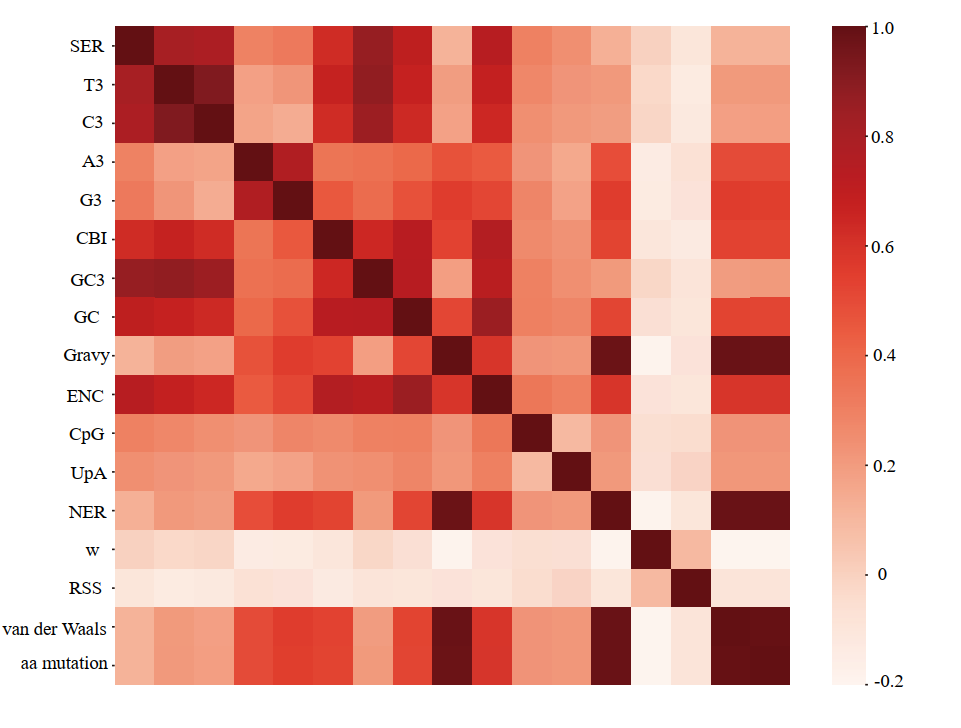


**Figure S5.** Spearman correlation coefficient symmetric heatmap between continuous features and SER.

**Supplementary Tables**

**Table S1 ORF lengths for SARS-CoV-2 genome**

| ORFs | Length(bp) |
| --- | --- |
| ORF1a | 13203 |
| ORF1b | 8085 |
| S | 3819 |
| ORF3a | 825 |
| E | 225 |
| M | 666 |
| ORF6 | 183 |
| ORF7a | 363 |
| ORF7b | 129 |
| ORF8 | 363 |
| N | 1257 |
| ORF10 | 114 |

**Table S2. Specific position and its ORF in SARS-CoV-2 constrained region**

| Position | ORF |
| --- | --- |
| 1306:1551 | ORF1a |
| 2773:3679 | ORF1a |
| 4279:4501 | ORF1a |
| 8926:10336 | ORF1a |
| 15061:15199 | ORF1b |
| 16060:16384 | ORF1b |
| 20005:20359 | ORF1b |
| 21289:21340 | ORF1b |
| 22720:22738 | S protein |
| 23458:23989 | S protein |

**Table S3. Contributing features used in this study**

| Index | Description |
| --- | --- |
| Codon Usage Index | |
| A3 | The content of A in the third codon position |
| T3 | The content of T in the third codon position |
| G3* | The content of G in the third codon position |
| C3 | The content of C in the third codon position |
| CBI | Codon bias index, it measures the extent to which a gene uses a subset of optimal codons.  CBI = 1.0, extreme codon bias; CBI = 0.0, random codon usage. |
| GC3 | GC content at the third codon position |
| GC | G+C content of the gene |
| ENC | Effective number of codons. The reported value of Nc is always between 20 (when only one codon is effectively used for each amino acid) and 61 (when codons are used randomly). |
| Dinucleotide Composition | |
| UpA | UpA dinucleotide enrichment degree in gene |
| CpG | CpG dinucleotide enrichment degree in gene |
| Selection Index | |
| w | dN/dS value in ORFs |
| NER | Non-synonymous evolutionary rate (NER) for each window |
| Structure Index | |
| RSS | RNA Secondary Structure, the minimum free energy of RNA secondary structure |
| Gravy* | The sum of hydropathy values of all amino acids divided by the protein length |
| van der Waals* | Normalized van der Waals volume |
| aa mutation* | Amino acid relative mutability |
| Conserved motifs | Motif1-Motif10 |

* Features excluded due to collinearity.

**Table S4. Model hyperparameters for LightGBM model**

| Hyperparameters | value |
| --- | --- |
| Learning rate | 0.03 |
| N estimators | 200 |
| Number leaves | 50 |
| Reg_lambda | 0.5 |
| Random state | 1000 |

**Table S5. LightGBM model performance**

| Index | Training set | Test set |
| --- | --- | --- |
| Mean square error | 6.46e-5 | 6.80e-5 |
| Mean absolute error | 5.89e-5 | 5.89e-5 |
| R2 | 0.72 | 0.69 |
| Adjusted R2 | 0.72 | 0.69 |
