# Supplementary material for: Variation in synonymous evolutionary rates in the SARS-CoV-2 genome
Source: Front Microbiol. 2023 Mar 9;14:1136386. doi: 10.3389/fmicb.2023.1136386 (PMC10034387; doi:10.3389/fmicb.2023.1136386)
Supplement: Supplementary file 3 [file Table_2.PDF]

SUPPLEMENTAL TABLE

Data Availability

GISAID Identifier:	EPI_SET_230130ru
doi:	10.55876/gis8.230130ru

All genome sequences and associated metadata in this dataset are published in GISAID's EpiCoV database. To view the contributors of each individual sequence with details such as accession number, Virus name, Collection date, Originating Lab and Submitting Lab and the list of Authors, visit 10.55876/gis8.230130ru

Data Snapshot

- EPI_SET_230130ru is composed of 10,084 individual genome sequences.
- The collection dates range from 2019-12-24 to 2021-09-10;
- Data were collected in 170 countries and territories;
- All sequences in this dataset are compared relative to hCoV-19/Wuhan/WIV04/2019 (WIV04), the official reference sequence employed by GISAID (EPI_ISL_402124). Learn more at https://gisaid.org/WIV04.

```
            1
EPI_ISL_2824951  ATGGAGAGCC TTGTCCCTGG TTTCAACGAG AAAACACACG TCCAACTCAG TTTGCCTGTT
EPI_ISL_3708868  ATGGAGAGCC TTGTCCCTGG TTTCAACGAG AAAACACACG TCCAACTCAG TTTGCCTGTT
EPI_ISL_1145037  ATGGAGAGCC TTGTCCCTGG TTTCAACGAG AAAACACACG TCCAACTCAG TTTGCCTGTT
EPI_ISL_1683896  ATGGAGAGCC TTGTCCCTGG TTTCAACGAG AAAACACACG TCCAACTCAG TTTGCCTGTT
EPI_ISL_2631277  ATGGAGAGCC TTGTCCCTGG TTTCAACGAG AAAACACACG TCCAACTCAG TTTGCCTGTT

           61
EPI_ISL_2824951  TTACAGGTTC GCGACGTGCT CGTACGTGGC TTTGGAGACT CCGTGGAGGA GGTCTTATCA
EPI_ISL_3708868  TTACAGGTTC GCGACGTGCT CGTACGTGGC TTTGGAGACT CCGTGGAGGA GGTCTTATCA
EPI_ISL_1145037  TTACAGGTTC GCGACGTGCT CGTACGTGGC TTTGGAGACT CCGTGGAGGA GGTCTTATCA
EPI_ISL_1683896  TTACAGGTTC GCGACGTGCT CGTACGTGGC TTTGGAGACT CCGTGGAGGA GGTCTTATCA
EPI_ISL_2631277  TTACAGGTTC GCGACGTGCT CGTACGTGGC TTTGGAGACT CCGTGGAGGA GGTCTTATCA

          121
EPI_ISL_2824951  GAGGCACGTC AACATCTTAA AGATGGCACT TGTGGCTTAG TAGAAGTTGA AAAAGGCGTT
EPI_ISL_3708868  GAGGCACGTC AACATCTTAA AGATGGCACT TGTGGCTTAG TAGAAGTTGA AAAAGGCGTT
EPI_ISL_1145037  GAGGCACGTC AACATCTTAA AGATGGCACT TGTGGCTTAG TAGAAGTTGA AAAAGGCGTT
EPI_ISL_1683896  GAGGCACGTC AACATCTTAA AGATGGCACT TGTGGCTTAG TAGAAGTTGA AAAAGGCGTT
EPI_ISL_2631277  GAGGCACGTC AACATCTTAA AGATGGCACT TGTGGCTTAG TAGAAGTTGA AAAAGGCGTT

          181
EPI_ISL_2824951  TTGCCCCAAC TTGAACAGCC CTATGTGTTC ATCAAACGTT CGGATGCTCG AACTGCACCT
EPI_ISL_3708868  TTGCCTCAAC TTGAACAGCC CTATGTGTTC ATCAAACGTT CGGATGCTCG AACTGCACCT
EPI_ISL_1145037  TTGCCTCAAC TTGAACAGCC CTATGTGTTC ATCAAACGTT CGGATGCTCG AACTGCACCT
EPI_ISL_1683896  TTGCCTCAAC TTGAACAGCC CTATGTGTTC ATCAAACGTT CGGATGCTCG AACTGCACCT
EPI_ISL_2631277  TTGCCTCAAC TTGAACAGCC CTATGTGTTC ATCAAACGTT CGGATGCTCG AACTGCACCT

          241
EPI_ISL_2824951  CATGGTCATG TTATGGTTGA GCTGGTAGCA GAACTCGAAG GCATTCAGTA CGGTCGTAGT
EPI_ISL_3708868  CATGGTCATG TTATGGTTGA GCTGGTAGCA GAACTCGAAG GCATTCAGTA CGGTCGTAGT
EPI_ISL_1145037  CATGGTCATG TTATGGTTGA GCTGGTAGCA GAACTCGAAG GCATTCAGTA CGGTCGTAGT
EPI_ISL_1683896  CATGGTCATG TTATGGTTGA GCTGGTAGCA GAACTCGAAG GCATTCAGTA CGGTCGTAGT
EPI_ISL_2631277  CATGGTCATG TTATGGTTGA GCTGGTAGCA GAACTCGAAG GCATTCAGTA CGGTCGTAGT

          301
EPI_ISL_2824951  GGTGAGACAC TTGGTGTCCT TGTCCCTCAT GTGGGCGAAA TACCAGTGGC TTACCGCAAG
EPI_ISL_3708868  GGTGAGACAC TTGGTGTCCT TGTCCCTCAT GTGGGCGAAA TACCAGTGGC TTACCGCAAG
EPI_ISL_1145037  GGTGAGACAC TTGGTGTCCT TGTCCCTCAT GTGGGCGAAA TACCAGTGGC TTACCGCAAG
EPI_ISL_1683896  GGTGAGACAC TTGGTGTCCT TGTCCCTCAT GTGGGCGAAA TACCAGTGGC TTACCGCAAG
EPI_ISL_2631277  GGTGAGACAC TTGGTGTCCT TGTCCCTCAT GTGGGCGAAA TACCAGTGGC TTACCGCAAG

          361
EPI_ISL_2824951  GTTCTTCTTN GTAAGAACGG TAATAAAGGA GCTGGTGGCC ATAGTTACGG CGCCGATCTA
EPI_ISL_3708868  GTTCTTCTTN GTAAGAACGG TAATAAAGGA GCTGGTGGCC ATAGTTACGG CGCCGATCTA
EPI_ISL_1145037  GTTCTTCTTN GTAAGAACGG TAATAAAGGA GCTGGTGGCC ATAGTTACGG CGCCGATCTA
EPI_ISL_1683896  GTTCTTCTTN GTAAGAACGG TAATAAAGGA GCTGGTGGCC ATAGTTACGG CGCCGATCTA
EPI_ISL_2631277  GTTCTTCTTN GTAAGAACGG TAATAAAGGA GCTGGTGGCC ATAGTTACGG CGCCGATCTA

          421
EPI_ISL_2824951  AAGTCATTTG ACTTAGGCGA CGAGCTTGGC ACTGATCCTT ATGAAGATTT TCAAGAAAAC
EPI_ISL_3708868  AAGTCATTTG ACTTAGGCGA CGAGCTTGGC ACTGATCCTT ATGAAGATTT TCAAGAAAAC
EPI_ISL_1145037  AAGTCATTTG ACTTAGGCGA CGAGCTTGGC ACTGATCCTT ATGAAGATTT TCAAGAAAAC
EPI_ISL_1683896  AAGTCATTTG ACTTAGGCGA CGAGCTTGGC ACTGATCCTT ATGAAGATTT TCAAGAAAAC
EPI_ISL_2631277  AAGTCATTTG ACTTAGGCGA CGAGCTTGGC ACTGATCCTT ATGAAGATTT TCAAGAAAAC

          481
EPI_ISL_2824951  TGGAACACTA AACATAGCAG TGGTGTTACC CGTGAACTCA TGCGTGAGCT TAACGGAGGG
EPI_ISL_3708868  TGGAACACTA AACATAGCAG TGGTGTTACC CGTGAACTCA TGCGTGAGCT TAACGGAGGG
EPI_ISL_1145037  TGGAACACTA AACATAGCAG TGGTGTTACC CGTGAACTCA TGCGTGAGCT TAACGGAGGG
EPI_ISL_1683896  TGGAACACTA AACATAGCAG TGGTGTTACC CGTGAACTCA TGCGTGAGCT TAACGGAGGG
EPI_ISL_2631277  TGGAACACTA AACATAGCAG TGGTGTTACC CGTGAACTCA TGCGTGAGCT TAACGGAGGG

          541
EPI_ISL_2824951  GCATACACTC GCTATGTCGA TAACAACTTC TGTGGCCCTG ATGGCTACCC TCTTGAGTGC
EPI_ISL_3708868  GCATACACTC GCTATGTCGA TAACAACTTC TGTGGCCCTG ATGGCTACCC TCTTGAGTGC
EPI_ISL_1145037  GCATACACTC GCTATGTCGA TAACAACTTC TGTGGCCCTG ATGGCTACCC TCTTGAGTGC
EPI_ISL_1683896  GCATACACTC GCTATGTCGA TAACAACTTC TGTGGCCCTG ATGGCTACCC TCTTGAGTGC
EPI_ISL_2631277  GCATACACTC GCTATGTCGA TAACAACTTC TGTGGCCCTG ATGGCTACCC TCTTGAGTGC

          601
EPI_ISL_2824951  ATTAAAGACC TTCTAGCACG TGCTGGTAAA GCTTCATGCA CTTTGTCCGA ACAACTGGAC
EPI_ISL_3708868  ATTAAAGACC TTCTAGCACG TGCTGGTAAA GCTTCATGCA CTTTGTCCGA ACAACTGGAC
EPI_ISL_1145037  ATTAAAGACC TTCTAGCACG TGCTGGTAAA GCTTCATGCA CTTTGTCCGA ACAACTGGAC
EPI_ISL_1683896  ATTAAAGACC TTCTAGCACG TGCTGGTAAA GCTTCATGCA CTTTGTCCGA ACAACTGGAC
EPI_ISL_2631277  ATTAAAGACC TTCTAGCACG TGCTGGTAAA GCTTCATGCA CTTTGTCCGA ACAACTGGAC
```
